# Supplementary material for: Effects of transcranial magnetic stimulation over the left posterior superior temporal gyrus on picture-word interference
Source: PLoS One. 2020 Nov 30;15(11):e0242941. doi: 10.1371/journal.pone.0242941 (PMC7703954; doi:10.1371/journal.pone.0242941)
Supplement: S1 File — (DOCX) [file pone.0242941.s001.docx]

***Effects of transcranial magnetic stimulation over the left posterior superior temporal gyrus on picture-word interference***

**S1 Table.** Mean response time (RT) and standard deviation (SD) for each distractor, list, and stimulation conditions.

| Distractor | List | Stimulation | Mean RT | SD |
| --- | --- | --- | --- | --- |
| unrelated | 1 | Vertex | 832 | 106 |
| unrelated | 1 | pSTG | 849 | 105 |
| unrelated | 2 | Vertex | 885 | 113 |
| unrelated | 2 | pSTG | 866 | 110 |
| related | 1 | Vertex | 868 | 99 |
| related | 1 | pSTG | 877 | 112 |
| related | 2 | Vertex | 914 | 109 |
| related | 2 | pSTG | 899 | 120 |
| congruent | 1 | Vertex | 728 | 124 |
| congruent | 1 | pSTG | 766 | 115 |
| congruent | 2 | Vertex | 814 | 124 |
| congruent | 2 | pSTG | 751 | 118 |

**Error analysis of the TMS experiment**

Mean error rates are presented in Table S2. Table S3 presents the details of the statistics for the errors. For list 1, a congruency effect was found. For list 2, both semantic and congruency effects were significant.

**S2 Table.** **Error percentage.** Error percentage (and SD) for each distractor condition by stimulation site and stimulus list. pSTG = mid-to-posterior superior temporal gyrus.

|  | **List 1** | | **List 2** | |
| --- | --- | --- | --- | --- |
| **Distractor by stimulation** | Vertex | pSTG | Vertex | pSTG |
| unrelated | 3.31 (3.64) | 2.31 (2.48) | 1.45 (1.78) | 3.17 (4.08) |
| related | 3.45 (4.72) | 3.75 (2.84) | 4.08 (2.79) | 3.9 (5.67) |
| congruent | 0 (0) | 0.722 (1.16) | 0.291 (0.67) | 1.3 (1.54) |

**S3 Table.** **Inferential statistics for the error rates.** The logistic regression model included by-participant random intercepts. SE = standard error; pSTG = mid-to-posterior superior temporal gyrus.

| **Condition / parameter** | **Odds Ratio** | **SE** | **z** | **p** |
| --- | --- | --- | --- | --- |
| List 1 |  |  |  |  |
| (Intercept) | 84.73 | 0.25 | 17.92 | <0.001 |
| Unrelated (vs related) | 1.30 | 0.21 | 1.23 | 0.220 |
| Congruent (vs unrelated) | 8.13 | 0.47 | 4.50 | <0.001 |
| pSTG stimulation (vs vertex) | 0.88 | 0.40 | -0.33 | 0.744 |
| List 2 | 0.653 | 0.517 | 1.263 | 0.207 |
| (Intercept) | 76.49 | 0.24 | 18.10 | <0.001 |
| Unrelated (vs related) | 1.93 | 0.23 | 2.81 | 0.005 |
| Congruent (vs unrelated) | 3.58 | 0.43 | 2.99 | 0.003 |
| pSTG stimulation (vs vertex) | 0.55 | 0.46 | -1.30 | 0.194 |
| Unrelated (vs related): pSTG vs vertex | 0.44 | 0.47 | -1.78 | 0.074 |
| Congruent (vs unrelated): pSTG vs vertex | 0.50 | 0.85 | -0.82 | 0.410 |

Figure S1 shows the relationship between participants’ mean response time (in seconds) and mean accuracy (in percentage) for each distractor condition (related, unrelated, congruent) per simulation site (Vertex, pSTG) and list (1, 2). Spearman’s correlation coefficients (and the corresponding p-values) are shown for each scatterplot. None of the correlations were significant (all *p*s > 0.220). Moreover, we ran a linear regression for the congruent condition in list 2 separately, with mean RT as the dependent variable and mean accuracy in interaction with stimulation site as predictors. The results of this analysis are shown in Table S4. None of the predictors were significant in the model. Thus, there is no indication of a speed-accuracy trade-off in the data.


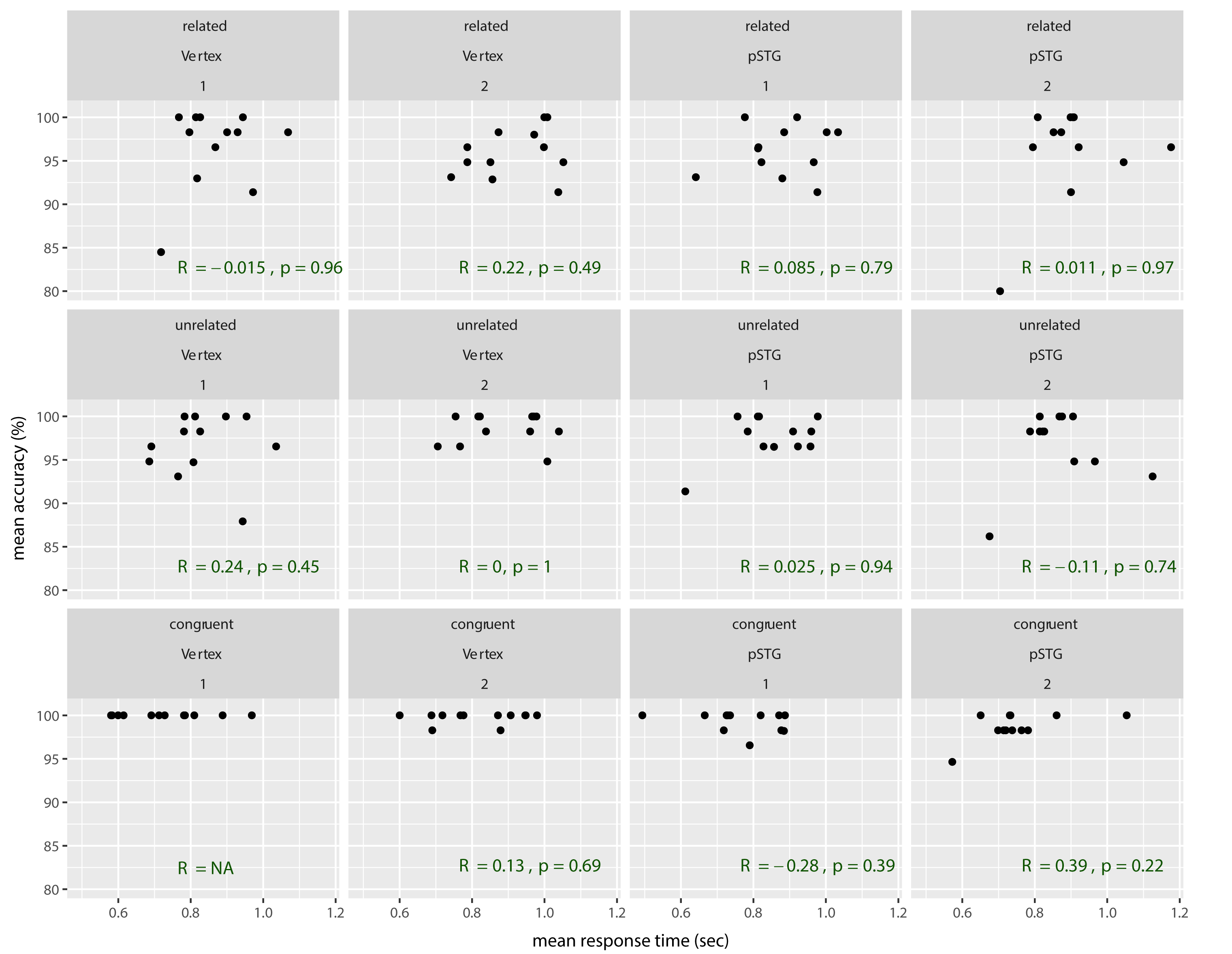


**S1 Figure.** Scatterplots of the relationship between participants’ mean response time (in seconds) and mean accuracy (in percentage) for each distractor condition (related, unrelated, congruent) per simulation site (Vertex, pSTG) and list (1, 2). Each dot represents one participant. Spearman’s correlation coefficients and corresponding p values are shown in green.

**S4 Table.** Inferential statistics for the relationship between mean response time and mean accuracy and their interaction.

| *Predictors* | *estimates* | *std. error* | *t statistic* | *p* |
| --- | --- | --- | --- | --- |
| Intercept | -2.38 | 2.84 | -0.84 | 0.412 |
| Mean accuracy | 0.03 | 0.03 | 1.12 | 0.276 |
| Stimulation site | -2.23 | 5.68 | -0.39 | 0.699 |
| Mean accuracy * stimulation site | 0.02 | 0.06 | 0.39 | 0.701 |
